# Host Selectivity and Distribution of *Cassytha filiformis* in the Coastal Bornean Heath Forests

**DOI:** 10.21315/tlsr2024.35.2.1

**Published:** 2024-07-31

**Authors:** Roshanizah Rosli, Kushan U. Tennakoon, Muhammad Yusran S. M. Yaakub, Nur Aqilah H. Zainal Ariffin, Faizah Metali

**Affiliations:** 1 Environmental and Life Sciences Programme, Faculty of Science, Universiti Brunei Darussalam, Jalan Tungku Link, BE1410 Gadong, Brunei Darussalam; 2 Institute for Biodiversity and Environmental Research, Universiti Brunei Darussalam, Jalan Tungku Link, BE1410 Gadong, Brunei Darussalam; 3 Institute of Innovation, Science and Sustainability, Future Regions Research Centre, Federation University, Berwick Campus, 100 Clyde Rd, Berwick VIC 3806, Australia

**Keywords:** *Cassytha filiformis*, Hemiparasites, Heath Forests, Haustoria, Host Selectivity

## Abstract

We investigated the host range of *Cassytha filiformis* L. in the heath forests using six 50-metre transects. Sixteen shrubs and tree species were infected by *C. filiformis* vines, including two exotic *Acacia* species. This paper also examined the density and vigour of *C. filiformis* when infecting the two most preferred and common hosts, the heath native *Dillenia suffruticosa* (Griff. ex Hook. f. and Thomson) Martelli, and the invasive *Acacia mangium* Willd. The results suggested that *C. filiformis* has higher vigour when infecting native hosts than in exotic *A. mangium* albeit being not statistically significant. The long thread-like stems of parasite were present at relatively high density when infecting *A. mangium*, regardless of the host conditions. We also assessed the functionality of the haustoria on both *D. suffruticosa* and *A. mangium* using histological methods. It was found that *C. filiformis* can establish a true haustorial endophytic connection with studied hosts. Under controlled conditions, *C. filiformis* pose as a possible candidate for a biological control agent of *A. mangium* to curtail the fast spreading of this introduced species in tropical Borneo.

**Highlights**

- The investigation of the host selectivity of *Cassytha filiformis* in the heath forests using six 50-meter transects revealed that sixteen shrubs and tree species were infected by the parasitic vines, including two exotic *Acacia* species.
- *C. filiformis* exhibited higher vigour when infecting native hosts compared to exotic *A. mangium* and demonstrated relatively high density when infecting *A. mangium*, irrespective of host conditions.
- Using histological methods, *C. filiformis* can establish a true haustorial endophytic connection with *A. mangium* and *D. suffruticosa*.

## INTRODUCTION

Throughout the course of evolutionary transitions, about 1% of angiosperms (Westwood *et al*. 2010) have adapted parasitism by acquiring resources from other plants via specialised organs of a morphological and physiological function called haustoria (Kuijt 1969; Yoshida *et al*. 2016; Teixeira-Costa & Davis 2021). Parasitic plants are often categorised by the extent of their host dependence (Heide-Jørgensen 2008). Facultative parasites are known to survive without a host for a certain period but would obtain their supply of water and/or nutrients when the opportunity arises. Alternatively, there are those that must require a host to live which are referred to as obligate parasitic plants. These plants are also recognised for their ability to photosynthesise (hemiparasites) or entirely non-chlorophyllous (holoparasites) (Musselman & Press 1995).

In terms of host preference, except for a few other specialists, most parasitic plants have a broad host range, especially when occurring in their natural habitat (Nickrent 2002). However, host specificity and the choice of hosts to infect ultimately depend on its accessibility and ability to locate hosts by selectively spreading towards or away from hosts, or by selectively penetrating host tissues upon contact through haustoria (Callaway & Pennings 1998; Runyon *et al*. 2006; Marquardt & Pennings 2010; Facelli *et al*. 2020).

*Cassytha* of the subfamily Cassythoideae is the only parasitic genus in the Lauraceae family (Awang *et al*. 2018). *Cassytha filiformis* Mill. is the sole pantropical species with wide global distribution in Asia, Africa, and tropical and subtropical America (Sastri 1962). It is a perennial hemiparasitic vine that infects its hosts by attaching to their stems. The generalist *Cassytha* has a relatively large and well-documented host range (Zhang *et al*. 2022). Despite the availability of hosts in the field, the obligate *C. filiformis* strands are often seen parasitising on only certain host species thus demonstrating the parasites’ preferential behaviour as highlighted by Koch *et al*. (2004) and Facelli *et al*. (2020). A common trait among generalists, the varying level of infection is also an indication of the mechanism of either active parasitism or a possible resistance on hosts (Kelly 1992) which could be examined by investigating the host stem histology and its anatomical response to the penetrating haustoria (Zhang *et al*. 2022). For instance, in a study by Facelli *et al*. (2020), *Acacia myrtifolia* was reported to exhibit resistance against the infection of *Cassytha pubescens* despite the presence of a firmly attached haustorium due to the lack of developed vascular connections. Under histological methods, the thickening cortical tissue of native species *A. myrtifolia* was observed thus preventing the parasite from forming true functional haustoria.

*Cassytha* are often seen along the coasts, sprawling on various host species at beaches around the world (Furuhashi *et al*. 2016). This is also a typical occurrence in Brunei where *C. filiformis* is abundant along the coasts (Rosli 2014; Tennakoon *et al*. 2016). Other than the preliminary list of hosts from an opportunistic field survey by Tennakoon *et al*. (2016), the study of host specificity in *C. filiformis* is lacking in Southeast Asia.

Despite accounting for 1% of the country’s forest cover, most of tropical Brunei’s coastlines are covered by a characteristic forest type known as heath forest. Bornean heath forests, locally referred to as *Kerangas* which means an area where rice cannot grow in the native Iban language (Davies & Salim 1999; Jambul *et al*. 2020), are mainly attributed to the highly acidic and low nutrient soils, and often inhabited by plant species with unique adaptive features (Newbery 1991; Wong *et al*. 2015; Hattori *et al*. 2019).

Tropical heath forests, especially those in Borneo, are susceptible to drastic environmental changes and anthropogenic activities (Din *et al*. 2015; Jambul *et al*. 2020). Similarly in Brunei, drastic changes in the ecosystem in the last 30 years have altered the soil properties causing this unique forest to be sensitive to degradation, fire, and habitat fragmentation (Zoletto & Cicuzza 2022). This is further exacerbated by the subsequent growth of the invasive and exotic *Acacia* species (Jaafar *et al*. 2016; Tuah *et al*. 2020) resulting in the secondary development of the now-threatened tropical heath (*Kerangas)* forest.

Much of the current host, *C. filiformis* studies looked into areas of its bioactivity (e.g., Abubacker *et al*. 2005; Armenia *et al*. 2015; Agbodjento *et al*. 2020; Umedum *et al*. 2020), physiology (e.g., Mukhtar *et al*. 2010; Mahadevan & Jayasuriya 2013; Balasubramanian *et al*. 2014; Furuhashi *et al*. 2021) and phylogeny (Wu *et al*. 2017; Zhang *et al*. 2020), while there are only few that discussed the effect of the stem hemiparasite on different hosts of a particular ecosystem (Kokubugata & Yokota 2012; Prider *et al*. 2009; Cai *et al*. 2020).

We present the first study on the host selectivity of *C. filiformis* in the threatened tropical Bornean heath forests. We examined:

1. Host range parasitised by *C. filiformis* using the transect method.
2. The impact of infection on hosts’ vigour relative to the density and vigour of the hemiparasite stem strands.
3. The anatomy of the haustorial interface of selected hosts to determine its functionality.

## MATERIALS AND METHODS

### Study Site

The study was conducted in the secondary heath (*Kerangas*) forests along the coastal highway (from 4°57’59.99°N, 114°52’33.531°E to 4°59’6.22°N, 114°54’1.472°E), within ca. 5 km off the coast of Brunei Darussalam from July to August 2021. Heath forests in Borneo are characterised by aseasonal lowland tropical rainforests that develop predominately on podzolised, highly acidic, sandy soils with relatively low macronutrients (Ghazoul & Sheil 2010; Jaafar *et al*. 2016; Ibrahim *et al*. 2020). Brunei has a tropical equatorial climate with average temperatures of 25.5°C and 28.9°C during the night and day throughout the year and total rainfall of 3815.1 mm in 2021 (Brunei Meteorological Service, unpublished data).

**Figure 1:**
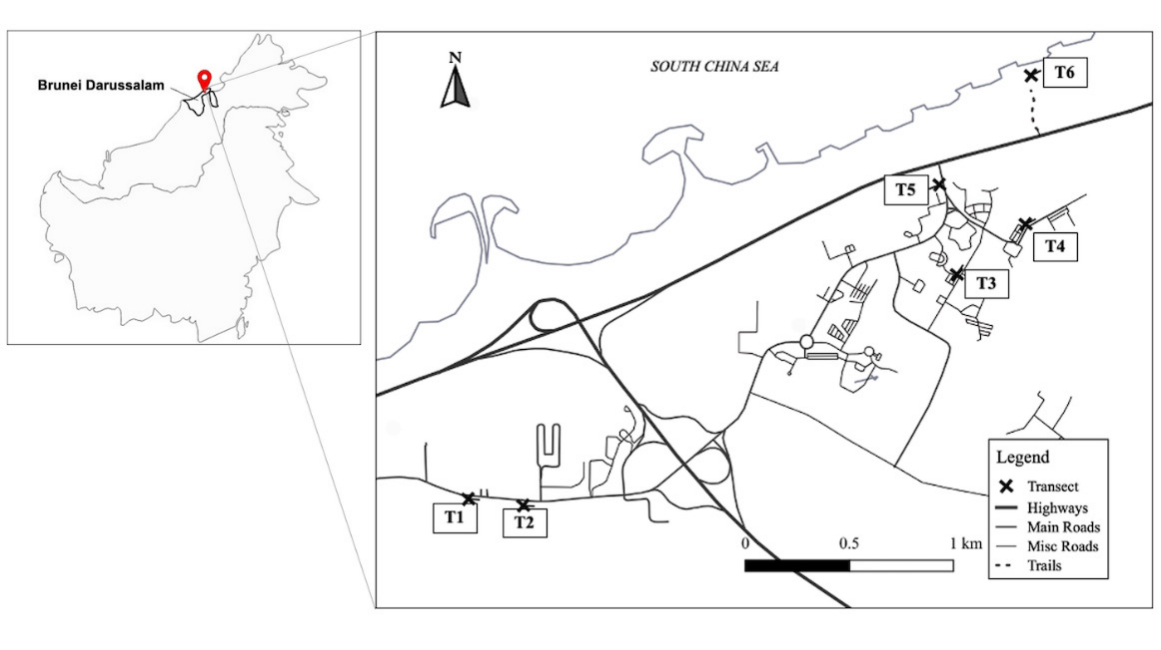
Map of Borneo Island (left) and the locations of the six transect surveys (T1 to T6) within the coastal heath forests of Brunei Darussalam (right).

In the study area, the secondary heath forests are inhabited by a co-occurring composition of native species, such as *Buchanania arborescens, Callophyllum inophyllum, Dillenia suffruticosa, Elaeocarpus mastersii, Melastoma malabathricum* and *Ploiarium alternifolium*, and the invasive species of *Acacia mangium, A. auriculiformis* and *A. holosericea* (Tuah *et al*. 2020). *C. filiformis* are also observed infecting certain host plants. These species exist within the vicinities of settlements and urban developments (Fig. 1; see also Jambul *et al*. 2020).

### Field Transect Survey

We established six 3 m × 50 m belt transects in July 2021 with ca. 0.5 m–1.0 m from the edge of the tropical heath forests. Within each transect, every individual of woody dicot species (i.e., shrubs and trees) with a height ca. 0.5 m and taller was recorded as “frequency of observation”, based on the methods employed by Kokubugata and Yokota (2012). The voucher specimens of the observed plants within the transect areas were collected for identification and confirmation at the Brunei National Herbarium (BRUN). Voucher specimens were deposited in the Universiti Brunei Darussalam Herbarium. To study the impact of infections on the two host plants with the highest frequencies of observations within these transects were selected, which are *Acacia mangium* Willd and *Dillenia suffruticosa* (Griff. ex Hook. f. and Thomson) Martelli.

*Acacia mangium* Willd. (hereafter *Acacia*) is a fast-growing leguminous tree species native to Australia and was introduced to Brunei in the late 1980s to mitigate soil erosion and as a timber plantation tree species (Osunkoya & Damit 2005; Ismail & Metali 2014; Jambul *et al*. 2020). It was then learnt that *Acacia* trees thrive in disturbed heath forests, especially since their seed dormancy is well-adapted to the recurring fire episodes and possesses the ability to fix nitrogen directly from the atmosphere (Jambul *et al*. 2020; Tuah *et al*. 2020). Osunkoya and Damit (2005) reported that *Acacia* could easily outcompete native plants such as *Melastoma beccarianum* under disturbed and degraded conditions, which eventually transform these habitats into nearly monospecific stands. *Dillenia suffruticosa* (Griff. ex Hook. f. and Thomson) Martelli. (hereafter *Dillenia*) is an important native pioneer shrub that may significantly impact the secondary succession of tropical forests (Rosli 2014). It is commonly distributed in disturbed areas, especially along roadsides and forest edges. Laboratory investigations have shown that *Dillenia* has anti-fungal, anti-bacterial and anti-cancer properties (Muliawan 2008; Armania *et al*. 2013; Goh *et al*. 2017).

The visual assessment of the host plants’ vigour and *Cassytha* cover were classified according to Prider *et al*. (2009). The vigour of *Cassytha* on each shrub was scored as “high” (actively growing, green stems), “low” (stems are partly dead and no active growth visible) or “dead” (no green stems). In our investigation, *Cassytha* cover was qualitatively scored as low, medium, high, and very high density. Low density infections covered <25% of the host where *Cassytha* was usually present as a few stems only, and medium density infections covered <50% of the host plant. High density infections covered <75% of the host, with *Cassytha* growing in entwined auto-parasitising strands to dense coiling mats. Very high density of *Cassytha* entailed the host plant being almost completely shrouded by the parasite, which can seem to deprive the hosts of sunlight.

Hosts’ growth condition or vigour was qualitatively scored as good, fair, poor and dead. “Good” hosts are when more than 90% of the individual plant is still alive where all or most of the leaves are green and intact. “Fair” host plants are 50% to 90% alive where some stems or leaves of hosts are dead or discoloured. Host plants that are mostly (<50%) dead or discoloured are scored as “poor”. Hosts are considered “dead” when all leaves are dead or discoloured. *Cassytha* infection was scored as present only when haustoria were observed on the plants within the transect areas. Chi-squared tests for independence were used to determine if there was a significant association between: (1) *Cassytha* vigour (i.e., High, Low, Dead) and its hosts; (2) *Cassytha* density (i.e., Very High, High, Medium, Low) and its hosts; (3) *Cassytha* density and the conditions of *Acacia* (i.e. Good, Fair, Poor, Dead); and (4) *Cassytha* density and the conditions of *Dillenia* (i.e., Good, Fair, Poor, Dead). Statistical analysis was conducted using R statistical programme version 4.1.3 RStudio (R Core Team 2022).

### Haustorial Anatomy

Developing and attached mature haustoria on the selected hosts, *Acacia* and *Dillenia*, were fixed in an ethanol and xylene series as described in Tennakoon and Cameron (2006) and embedded in wax blocks with the haustorial interface arranged longitudinally. Using a microtome (Shandon Finesse ME+ Thermo Electron Corporation, Cheshire, UK), 10 μm–20 μm thick sections were prepared and placed onto glass slides. The thickness of the sections was based on the hardness of the host stems. Young and soft host stems were preferable in this experiment to ease the microtome process. Waxed sections were de-waxed and rehydrated prior to staining with 1% Toluidine Blue. Histological sections were examined under a light microscope (Leica DM2500 Microsystems CMS GmbH, Wetzlar, Germany). Images were acquired using a digital camera (Olympus DP73, Tokyo, Japan) using CellSens imaging software (Version 1.9, Olympus, Tokyo, Japan).

## RESULTS

### Host-Parasite Associations

A total of 336 individual dicotyledonous plants (see Appendix) were sampled from the six transect areas, where 99 individuals (29.5%) were found infected (Table 1). A total of 17 species from 16 genera and 15 families were recorded as host species. *Buchanania arborescens, Dillenia suffruticosa, Elaeocarpus* aff. *mastersii, Nepenthes gracilis, Pouteria obovata, Psychotria sarmentosa, Rhodomyrtus tormentosa* and *Timonius flavescens* were the native heath or *Kerangas* species identified (Coode *et al*. 1996; Tuah *et al*. 2020). Two invasive, introduced plant species, *Acacia mangium* and *Acacia auriculiformis*, were common and frequently observed within the study sites. Other than these two, the host species in Table 1 are native to Brunei (Coode *et al*. 1996; Zamri & Slik 2018; Tuah *et al*. 2020) and they are common to secondary forests of Brunei (Coode *et al*. 1996).

**Figure 2:**
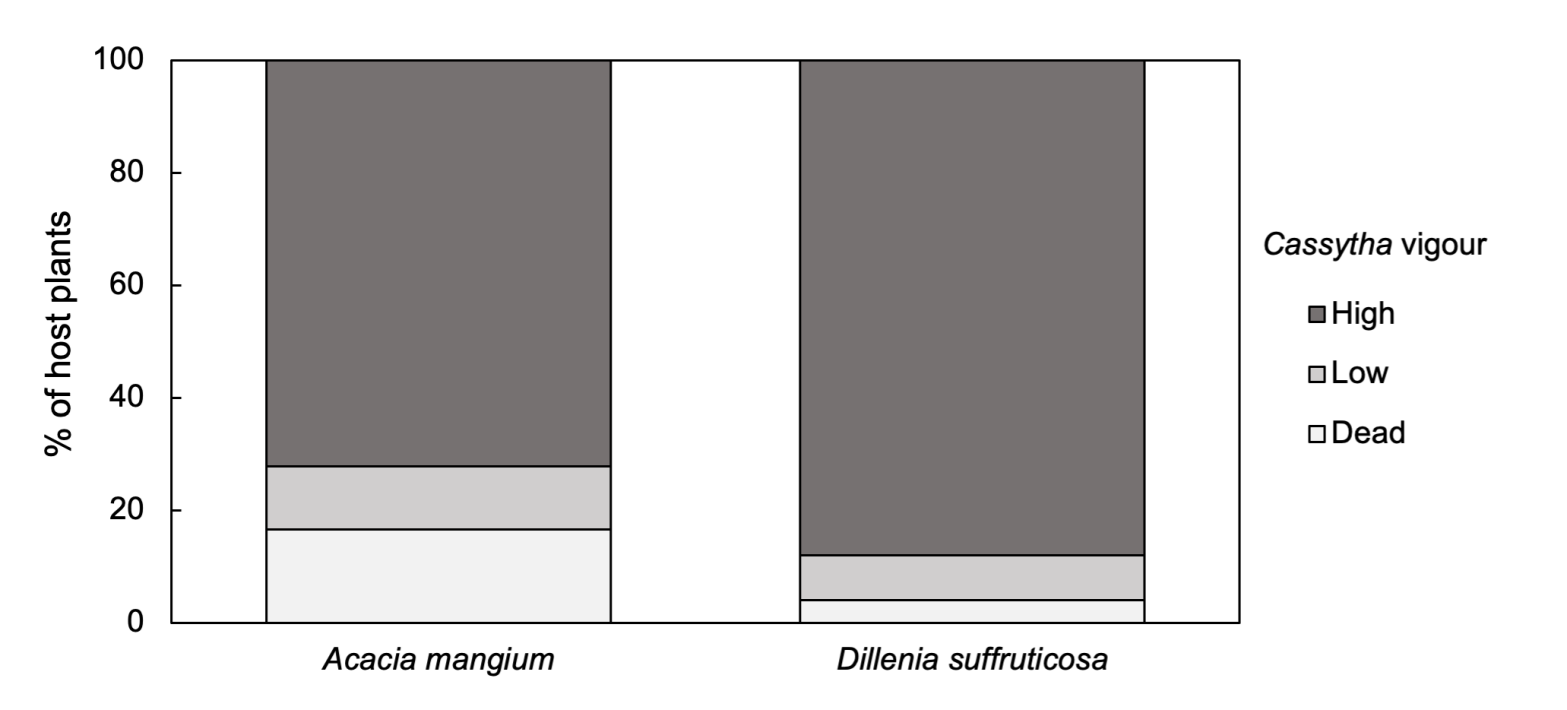
Impact of increasing *Cassytha filiformis* vigour on the two host species *Acacia mangium* and *Dillenia suffruticosa*. The vigour of *Cassytha* on each shrub was scored as “high” (actively growing, green stems), “low” (stems are partly dead and no active growth visible) or “dead” (no green stems).

*Dillenia* and *Acacia* were the two host plants with the highest frequency of observations (Table 1) and were selected to assess the *in-situ* effect of the *Cassytha* infection. The vigour of *Cassytha* while infecting the selected hosts was assessed in Fig. 2. *Cassytha* stems had higher vigour, i.e., better health while infecting the native *Dillenia* than that in *Acacia*, with more than 80% growing healthily in the former. It was found that there was higher mortality in *Cassytha* when infecting *Acacia* (16.7%) than that with *Dillenia* (4.0%). Chi-squared test was used to determine if there was a significant association between *Cassytha* vigour and the hosts. There was not a statistically significant association between the two variables (χ^2^(2, N = 43) = 2.24, *p* = 0.32).

**Figure 3:**
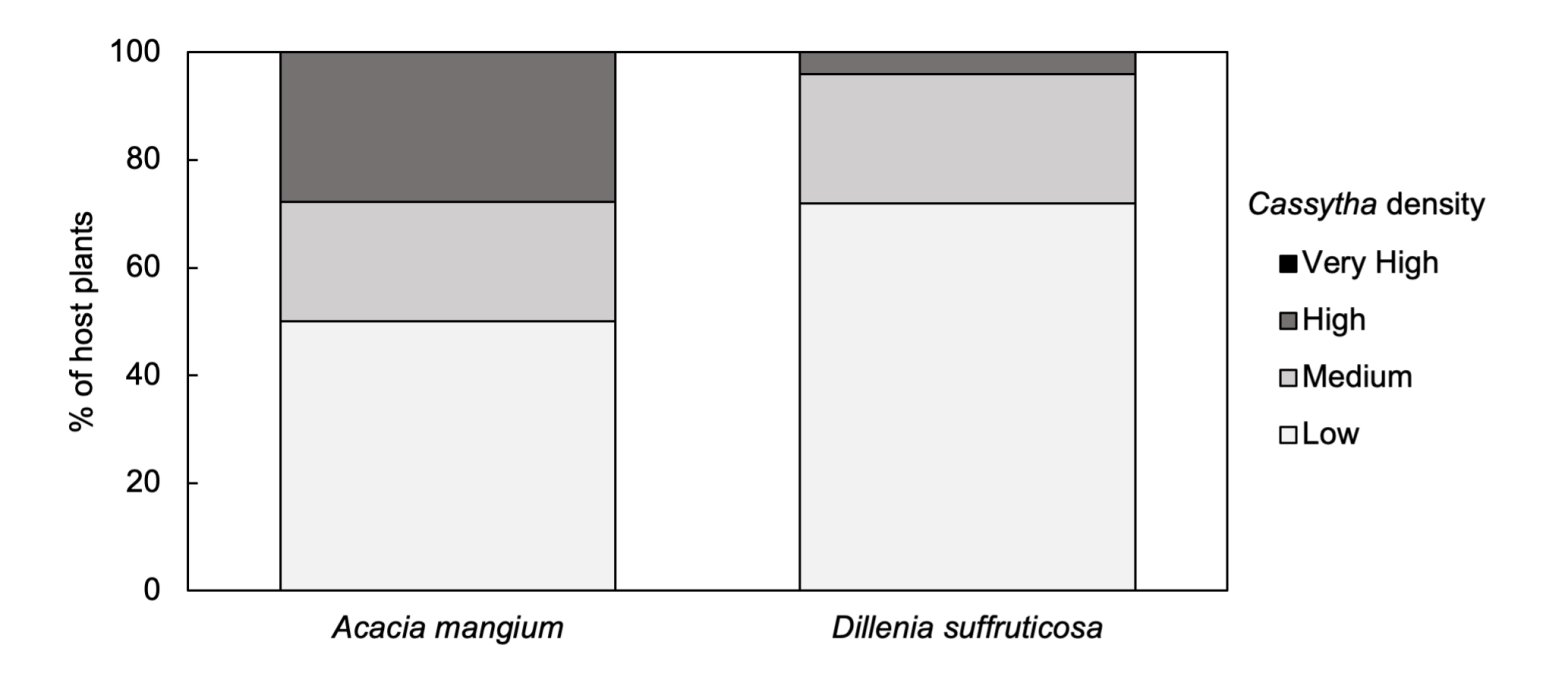
Impact of increasing *C. filiformis* densities on the two host species *A. mangium* and *D. suffruticosa*. *Cassytha* cover was qualitatively scored as low, medium, high and very high density. Low density infections covered <25% of the host where *Cassytha* was usually present as a few stems only, and medium density infections covered <50% of the host plant. High density infections covered <75% of the host, with *Cassytha* growing in entwined auto-parasitising strands to dense coiling mats. Very high density of *Cassytha* entailed the host plant being almost completely covered by the parasite.

The percentage of both host plants infected by various *Cassytha* densities is represented in Fig. 3. Despite the healthy growth of *Cassytha* on *Dillenia*, there was a higher infection density in the introduced species, with 27.8% and 22.2% of *Acacia* infected by high density and medium density of *Cassytha*, respectively. About 72% of *Dillenia* were infected by low density of *Cassytha*. None of the *Dillenia* and *Acacia* were infected by the very high density of *Cassytha*. Chi-squared test was also used to determine if there was a significant association between the increasing *Cassytha* density and the hosts. There was not a statistically significant association between the two variables (χ^2^ (2, N = 43) = 5.06, *p* = 0.08).

**Figure 4:**
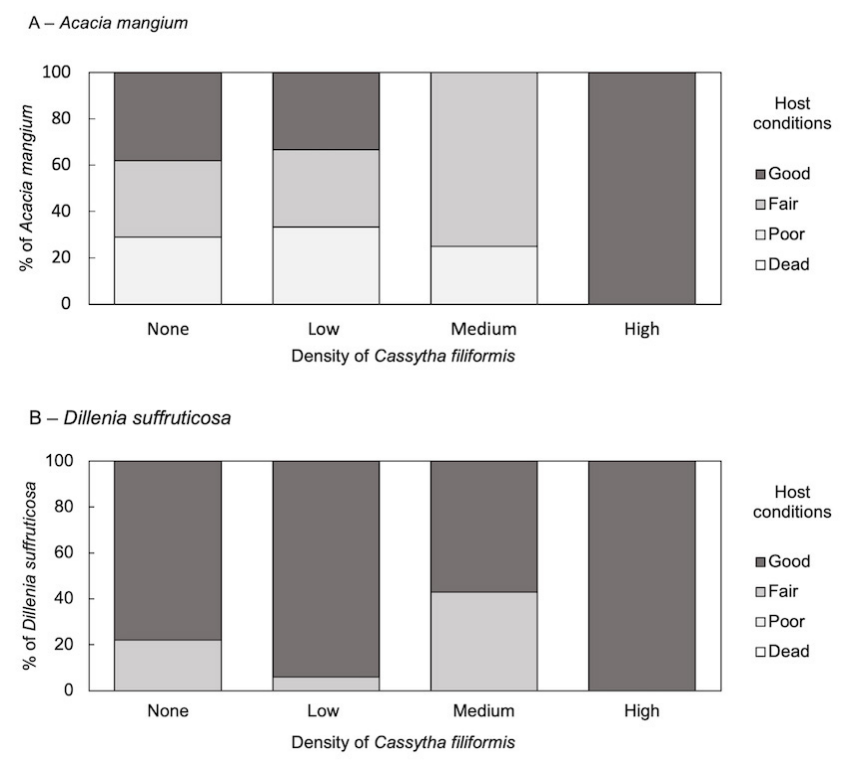
Frequency histograms of the proportions of: (A) *A. mangium*, and (B) *D. suffruticosa*, in different growth conditions when infected by *C. filiformis* of increasing density levels. Hosts’ growth condition or vigour was qualitatively scored as good, fair, poor and dead. “Good” hosts are when more than 90% of the individual plant is still alive where all or most of the leaves are green and intact. “Fair” host plants are 50% to 90% alive where some stem or leaves of hosts are dead or discoloured. Host plants that are mostly (<50%) dead or discoloured are scored as “poor”. Hosts are considered “dead” when all leaves are dead or discoloured.

Fig. 4 illustrates the health conditions or vigour of *Acacia mangium* and *Dillenia suffruticosa* with respect to the density of *C. filiformis* infection. In general, the virulence of *Cassytha* was high when host plants were healthy. However, the hemiparasite did not parasitise on *Dillenia* of lower vigour. Their preference was rather indifferent when infecting the introduced species where “poor” *Acacia* plants were parasitised by *Cassytha*. Chi-squared test was run to determine if there was a significant association between the increasing *Cassytha* densities and the growth conditions of hosts. There were no statistically significant associations between the two variables for both *Acacia* and *Dillenia* i.e., (χ^2^ (6, N = 94) = 11.69, *p* = 0.07) and (χ^2^ (3, N = 75) = 4.78, *p* = 0.19), respectively.

### Histology of Haustoria Formation

**Figure 5:**
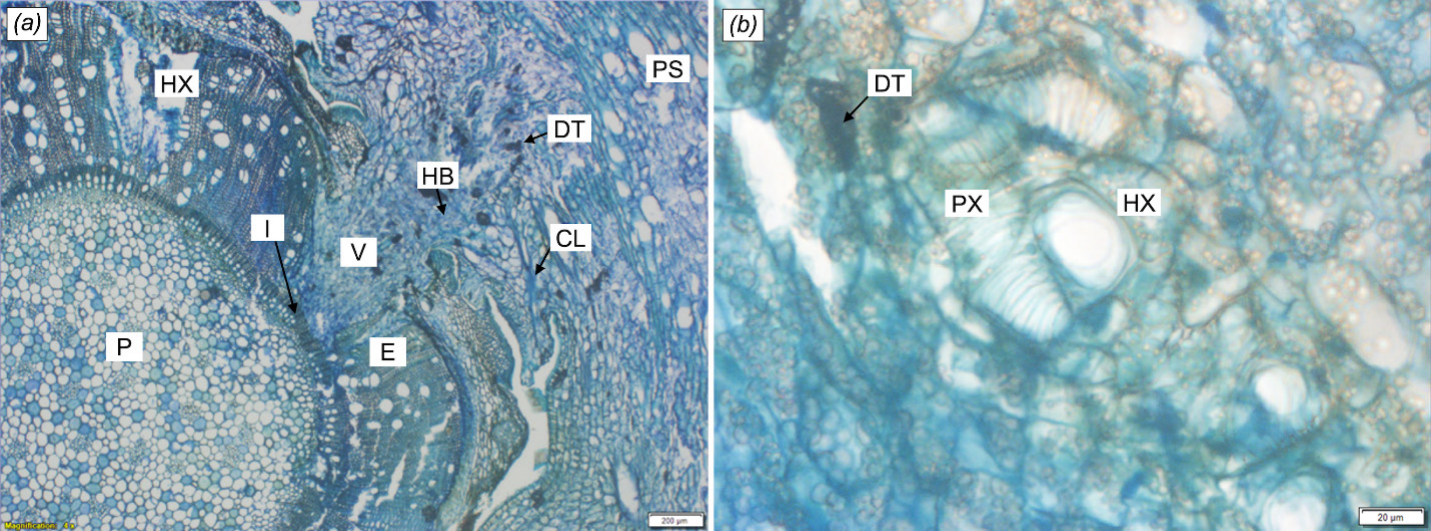
Detailed anatomy of the haustorial interface of *Cassytha filiformis* with *Dillenia suffruticosa* at (a) ×4 magnification, and (b) at ×40 magnification, highlighting direct lumen-to-lumen xylem connections between the xylems of the host (HX) and parasite (PX). H = haustoria; P = host stem pith; PS = parasite stem; E = endophyte; HX = host xylem; PX = parasite xylem; I = interface between host and parasite; V = vascular core; DT = darkly stained tissue; CL = collapsed layer; HB = hyaline body.

**Figure 6:**
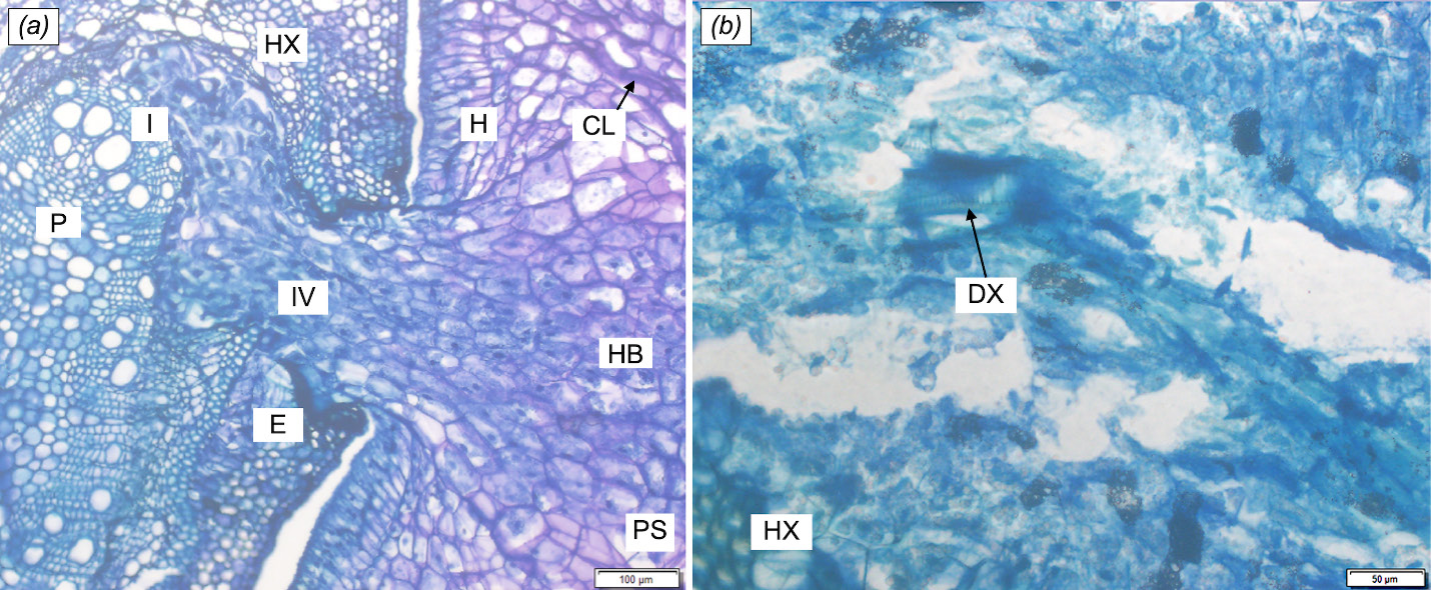
Detailed anatomy of the haustorial interface of *Cassytha filiformis* with *Acacia mangium* at (a) ×10 magnification (b) ×20 magnification, particularly a section of the haustorial endophyte. H = haustoria; P = host stem pith; PS = parasite stem; DX = differentiated xylem; E = endophyte; HX = host xylem; I = interface between host and parasite; IV = initial vascular core formation; CL = collapsed layer; HB = hyaline body.

Sections that were complete (intact) and best represent the behaviour of the haustoria are presented in Figs. 5 and 6. The haustorial endophytes had successfully reached and penetrated the vasculature of the host stems of *D. suffruticosa* (Fig. 5). The haustoria of *C. filiformis* appeared to grow into the host tissue in a wedge-like shape endophyte (E), mostly in direct contact with the host xylem (HX) (Fig. 5a). The presence of vascular core (V) was visible in middle section of the endophyte with the relatively high observation of xylem tissues. Another section of the same host – parasite (PX) association has shown direct luminal contact (Fig. 5b, PX, HX) with host xylem within the vascular core of the haustoria. Few cells of the endophytes were darkly stained, thus creating dense tissue (DT) in Fig. 5a. High nucleic-structures of hyaline body (HB) were present in the endophyte.

As with the haustoria of *C. filiformis* on *A. mangium* in Fig. 6, the endophyte seemed to have spread around the host vascular structure creating an ellipsoidal flattened disc increasing the surface area of contact (Fig. 6a, I). The mass differentiating parenchyma cells running through the middle section of the endophyte indicate the initial development of the vascular core (Fig. 6a, IV). While the initiation of vascular core is yet to be present in Fig. 6a, differentiated xylem (DX) within the endophyte is evident in a different histological section (Fig. 6b). The presence of HB is also visible. The wedge-like endophytic growth of the parasite within the host tissue is also observed in other haustorial sections. This may be due to the relative thickness of *D. suffruticosa* stems, i.e., ca. 1.5 cm in comparison to the stems of *A. mangium* (ca. 0.5 cm–1.0 cm).

## DISCUSSION

This study has shown the wide host specificity range of the hemiparasitic *C. filiformis*, thus exhibiting its generalist nature. This is evident in their unselective behaviour in infecting various host species, including the previously unrecorded grasses and fern species. The two most common host species for *C. filiformis* were *A. mangium* and *D. suffruticosa*. Although the parasite showed a slight preference for *Dillenia, Cassytha* thrives to high densities on both *Acacia* and *Dillenia*.

The results also demonstrated that under very high *Cassytha* density, a good *Acacia* stand exists. This may be because of the *in-situ* nature of the study where the age of the infection was not considered. The healthy *Acacia* was perhaps just newly infected, and the negative physiological effect of the infection was not apparent yet. Since every individual plant of height ca. 0.5 m and taller was recorded for this investigation, the age of the host plants is also highly variable. This potentially affects how the hosts respond to the parasitic infection. Nonetheless, host susceptibility to infection and the virulence of the parasite were greater in the introduced host than in the native host. This is a similar pattern observed in the parasitism of *Cassytha pubescens* on *Leptospermum myrsinoides* and *Cytisus scoparius*, a native and introduced species to Australia (Prider *et al*. 2009).

The soils of the threatened *Kerangas* forest are high in nitrogen and have always been negatively affected by *Acacia* trees which are invasive nitrogen-fixing legumes in Brunei (Tuah *et al*. 2020). A study by Yusoff *et al*. (2019) reported that there was a significantly higher concentration of total N in leaf litters in an *Acacia-*invaded *Kerangas* forest, suggesting that the invasive *Acacia* has further decreased the naturally poor soil nutrients. Non-fixers parasitic plants are likely to infect nitrogen-fixing hosts (Press *et al*. 1993; Seel & Press 1993) because hosts with high nutrient content such as legumes are often preferred (Matthies 1996; Pate & Bell 2000; Pennings & Callaway 2002) thus making *Acacia* spp. the highly favoured candidates as hosts. Recent nutrient studies on *Cassytha* hosts by Rosli *et al*. (unpublished data) have shown that *D. suffruticosa* has a similar amount of total N content (14.12 mg/g) to that of *A. mangium* (14.71 mg/g). The total N content of *D. suffruticosa* was also found to be higher than in the native pioneer, *Melastoma malabathricum* (12.59 mg/g).

The preference for hosts with relatively high nitrogen content is attributed to the lack of means to perform active uptake of such nutrients. Thus, hemiparasites like *Cassytha* opt to take organic nitrogen and other mineral nutrients that are diverted from the host xylem sap via the haustoria, to promote growth and increase their own biomass. Another reason for *Cassytha*’s acquisition of host-derived organic nitrogen elements is that they potentially lack the symbioses for nitrogenase enzyme production which is essential in biological nitrogen fixation. However, this assumption warrants further confirmation.

It is imperative to note that nitrogen-rich plants have reduced growth performance and are more vulnerable to parasitic infections which can further impair their stressed conditions (Kelly 1992; Gehring & Whitham 1992; Jeschke *et al*. 1994; Matthies 1996; Jeschke & Hilpert 1997; Pennings & Simpson 2008). Once infected, the host performance worsens as parasites thrive with the nutrients obtained from the hosts (Prider *et al*. 2009). It is evident in this study where *C. filiformis* also infected *A. mangium* at “poor” condition.

Bioactivity compounds attributed to the host-parasite dynamics also play a role in host specificity, specifically in the attachment process. The induction of chemical molecular signals, germination stimulants and haustoria-inducing factors are some examples of the products (Okubamichael *et al*. 2011; Yoshida *et al*. 2016). However, further investigations involving studies of bioactive compounds are required to confirm this potential cause.

Studies on host preference also reported that there are plant traits that appeared to be manipulated to demonstrate that they directly affect parasite preferences or performance (Kelly 1992; Pennings & Simpson 2008; Marquardt & Pennings 2010). This may account for *Dillenia* being one of the highest infected host plants in this study. This is also evident in the high percentage of *Dillenia* infection by a low density of *Cassytha*. *C. filiformis* are reported to prefer woody host plants with soft, thin barks and periderm and those with low and much-branched (Werth *et al*. 1979; Buriyo *et al*. 2015); both physical traits that are present in *Dillenia* as a pioneer, woody shrub that tends to grow in dense thickets. This indicates that *Dillenia* presents as a more accessible host to *Cassytha* by acquiring the required metabolites without investing much effort in heavy infestations.

Another possible explanation for the preferential behaviour may be attributed to the availability of more suitable resources which they acquire through the direct lumen-to-lumen linkages of the endophytes of *A. mangium* and *D. suffruticosa: Cassytha* associations. Through light microscopy investigations, this study was able to demonstrate such connections in the *Dillenia-Cassytha* association. This could not be captured in the *Acacia-Cassytha* sections, despite the proximity of the endophyte to the host vascular structure and the presence of the differentiating xylems. Thus, to further confirm this observation, we suggest utilising fluorescent trackers to the host root or sampling the xylem and phloem of the host and parasite and comparing the solute compositions (i.e., sugars, organic acids or amino acids) via isotope labelling (Tennakoon *et al*. 1997; Hibberd & Jeschke 2001; Jiang 2004; Tennakoon & Cameron 2006; Facelli *et al*. 2020).

Host tolerance to *Cassytha* infection may contribute to the reduced impacts of the parasites (Prider *et al*. 2009), however resistance was not observed in this study since no cases of pseudo-haustorial connections were encountered. It is also important to note that the field survey conducted in this *in situ* study did not determine if *Cassytha* was also connected to other surrounding hosts that could have been supporting its growth.

The outcomes of this study suggest that *C. filiformis* is indifferent to the hosts they parasitise, irrespective of whether hosts are native or exotic hosts. This confirms that generalist parasites are able to infect hosts which have not co-evolve to adopt a resistance or defence strategy (Koch *et al*. 2004; Cirocco *et al*. 2016). However, based on the results which highlight the higher density of *C. filiformis* on the invasive *A. mangium*, *C. filiformis* could be considered an important biological controlling agent under well-controlled conditions to reduce further spread of alien invasive *A. mangium* in tropical Southeast Asia. This concurs with the biotic resistance hypothesis where parasitic plants may be candidates for “a cost-effective environmentally sustainable component of invasion management scheme” (Těšitel *et al*. 2020). Generally, species that are used for biological control have high host specificity to ensure that only the targeted species is affected by the introduction of the species into a system (Myers & Bazely 2003). In the case of the secondary heath forest, most tree stands consist of the fast-growing *Acacia* species, and those infected are often in poor conditions based on field observations.

The next question should investigate this parasitism’s effects on the hosts’ physiology. The physiological impacts of parasites on invasive species have a greater effect on host health, biomass, and fecundity than on native hosts (Prider *et al*. 2009; Cirocco *et al*. 2016; 2018). Physiological studies such as photosynthetic activities and nutrient analysis on this host-parasitic association would be able to explain the extent of the impact of parasitism on these hosts within this unique site.

## CONCLUSION

*C. filiformis* exhibited low host-specificity with its wide range of hosts, irrespective of their nativity to the tropical heath habitat. This is illustrated in the well-established haustorial structures in both *A. mangium* and *D. suffruticosa*. However, employing better histological techniques, such as scanning electron microscopy (SEM), may illustrate detailed anatomical evidence to prove successful haustorial connections. Previous studies conducted on *C. filiformis* in Brunei suggested that the hemiparasitic vine has the potential to act as a biocontrol agent against invasive species. The outcome of this investigation has shown that even with high *Cassytha* vigour, infected hosts can still thrive and did not specifically fulfil the previous statement. This could also be a possible inkling of a co-existing behaviour of *Cassytha* to certain hosts. This would entail an intricate look at resistance genes in the host genomes. However, in the existing state of the heath forests in Brunei where natives are threatened to be outcompeted by the monodominant *A. mangium*, *C. filiformis* is a good candidate for a potential biocontrol agent. This is feasible under controlled conditions by careful monitoring and ensuring that the spread of the hemiparasitic vines is limited to the invasive *Acacia* species only.

Furthermore, there are several determining factors and experimental modifications to this study that could be included to further test the impact of *Cassytha* infection on these hosts such as host biomass and the environmental conditions, for example, *ex-situ* and greenhouse experiments where the growth of the parasites and their hosts are monitored. Nonetheless, the findings also indicate that *Cassytha* can still be used to reduce the spread of exotic weeds and invasive plants.

## Figures and Tables

**Table 1:** Summary of host plants from the six 3 m × 50 m transect surveys. The family and species names are arranged according to the frequencies of observations.

Host plants		Frequency of observation^a^

Family	Species	
Dilleniaceae	*Dillenia suffruticosa (*Griff. ex Hook.f. and Thomson) Martelli^b^	25
Fabaceae	*Acacia mangium* Willd^c^	19
Fabaceae	*Acacia auriculiformis* A. Cunn. ex Benth^c^	16
Melastomataceae	*Melastoma malabathricum* L.	10
Euphorbiaceae	*Endospermum diadenum* (Miq.) Airy Shaw	5
Nepenthaceae	*Nepenthes gracilis* Korth.^b^	5
Lamiaceae	*Vitex pinnata* L.	4
Elaeocarpaceae	*Elaeocarpus* aff. *mastersii* King^b^	3
Anacardiaceae	*Buchanania arborescens* (Blume) Blume^b^	2
Malvaceae	*Commersonia batramia* (L.) Merr.	2
Rubiaceae	*Timonius flavescens* (Jacq.) Baker^b^	2
Casuarinaceae	*Casuarina equisetifolia* L.	1
Euphorbiaceae	*Macaranga tanarius* (L.) Müll.Arg.	1
Myrtaceae	*Rhodomyrtus tomentosa* (Aiton) Hassk.^b^	1
Myrtaceae	*Syzygium acuminatissimum* (Blume) DC.^b^	1
Rubiaceae	*Psychotria sarmentosa* Blume^b^	1
Sapotaceae	*Pouteria obovata* (R.Br.) Baehni^b^	1

*Notes:*

a based on Kokubugata and Yokota (2012);

b native *Kerangas* species;

c introduced species.

## APPENDIX

List of 336 dicotyledonous individual plants that were sampled during the field survey at the six 50-m transects, noted with the host plants’ vigour and *Cassytha* density.[Table t2-tlsr-35-2-1]

**Table t2-tlsr-35-2-1:**

Site	Individual	Infected (/)	Host Plant Vigour	Cassytha Density	Genus	Species
T1	1	X	Good	-	*Acacia*	*mangium*
T1	2	X	Good	-	*Acacia*	*mangium*
T1	3	X	Good	-	*Acacia*	*mangium*
T1	4	X	Good	-	*Buchanania*	*arborescens*
T1	5	X	Good	-	*Acacia*	*mangium*
T1	6	X	Good	-	*Acacia*	*mangium*
T1	7	X	Good	-	*Acacia*	*mangium*
T1	8	X	Good	-	*Acacia*	*mangium*
T1	9	X	Good	-	*Acacia*	*auriculiformis*
T1	10	X	Good	-	*Acacia*	*auriculiformis*
T1	11	X	Good	-	*Acacia*	*mangium*
T1	12	X	Good	-	*Acacia*	*auriculiformis*
T1	13	X	Good	-	*Acacia*	*mangium*
T1	14	/	Good	High	*Acacia*	*auriculiformis*
T1	15	/	Good	High	*Buchanania*	*arborescens*
T1	16	/	Good	Low	*Syzygium*	*acuminatissimum*
T1	17	X	Good	-	*Syzygium*	*acuminatissimum*
T1	18	X	Good	-	*Callophylum*	*inophyllum*
T1	19	X	Good	-	*Acacia*	*mangium*
T1	20	/	Good	High	*Acacia*	*mangium*
T1	21	/	Good	Low	*Dilennia*	*suffruticosa*
T1	22	/	Good	Low	*Dilennia*	*suffruticosa*
T1	23	X	Good	-	*Acacia*	*mangium*
T1	24	/	Good	High	*Acacia*	*mangium*
T1	25	/	Good	Low	*Timonius*	*flavescens*
T1	26	/	Good	Low	*Acacia*	*auriculiformis*
T1	27	X	Good	-	*Syzygium*	*sp*.
T1	28	/	Good	High	*Acacia*	*mangium*
T1	29	/	Good	High	*Acacia*	*mangium*
T1	30	/	Good	Low	*Pouteria*	*obovata*
T1	31	/	Good	Low	*Endospermum*	*Diodenum*
T1	32	X	Good	-	*Endospermum*	*Diodenum*
T1	33	/	Good	Medium	*Endospermum*	*Diodenum*
T1	34	/	Good	Low	*Acacia*	*mangium*
T1	35	X	Good	-	*Acacia*	*mangium*
T1	36	/	Good	High	*Elaeocarpus*	*mastersii*
T1	37	/	Good	Medium	*Elaeocarpus*	*mastersii*
T1	38	/	Good	Medium	*Acacia*	*auriculiformis*
T1	39	/	Good	High	*Elaeocarpus*	*mastersii*
T1	40	/	Good	High	*Acacia*	*mangium*
T1	41	/	Good	Low	*Endospermum*	*diadenum*
T1	42	/	Good	Low	*Endospermum*	*diadenum*
T1	43	X	Good	-	*Elaeocarpus*	*mastersii*
T1	44	X	Good	-	*Endospermum*	*diadenum*
T1	45	X	Good	-	*Endospermum*	*diadenum*
T1	46	X	Good	-	*Acacia*	*auriculiformis*
T1	47	X	Good	-	*Endospermum*	*diadenum*
T1	48	X	Good	-	*Endospermum*	*diadenum*
T1	49	X	Good	-	*Endospermum*	*diadenum*
T1	50	X	Good	-	*Elaeocarpus*	*mastersii*
T1	51	/	Good	-	*Timonius*	*flavescens*
T1	52	X	Good	-	*Acacia*	*mangium*
T1	53	X	Good	-	*Pouteria*	*obovata*
T1	54	X	Good	-	*Pouteria*	*obovata*
T1	55	X	Good	-	*Endospermum*	*diadenum*
T1	56	X	Good	-	*Dillenia*	*suffruticosa*
T1	57	X	Good	-	*Pouteria*	*obovata*
T1	58	X	Good	-	*Licania*	*splendens*
T1	59	X	Good	-	*Elaocarpus*	*mastersii*
T1	60	X	Good	-	*Pouteria*	*obovata*
T1	61	X	Good	-	*Buchanania*	*arborescens*
T1	62	X	Good	-	*Acacia*	*mangium*
T1	63	X	Good	-	*Licania*	*splendens*
T1	64	X	Good	-	*Dilennia*	*suffruticosa*
T2	1	X	Poor	-	*Acacia*	*mangium*
T2	2	X	Poor	-	*Acacia*	*mangium*
T2	3	X	Poor	-	*Acacia*	*mangium*
T2	4	X	Poor	-	*Acacia*	*mangium*
T2	5	X	Poor	-	*Acacia*	*mangium*
T2	6	X	Fair	-	*Acacia*	*mangium*
T2	7	X	Fair	-	*Acacia*	*mangium*
T2	8	X	Good	-	*Syzygium*	*incarnatum*
T2	9	X	Good	-	*Acacia*	*mangium*
T2	10	X	Good	-	*Maranthes*	*corymbosa*
T2	11	X	Poor	-	*Acacia*	*mangium*
T2	12	X	Poor	-	*Acacia*	*mangium*
T2	13	X	Fair	-	*Acacia*	*mangium*
T2	14	X	Fair	-	*Acacia*	*mangium*
T2	15	X	Poor	-	*Acacia*	*mangium*
T2	16	X	Poor	-	*Acacia*	*mangium*
T2	17	X	Poor	-	*Acacia*	*mangium*
T2	18	/	Poor	Low	*Acacia*	*mangium*
T2	19	X	Fair	-	*Acacia*	*mangium*
T2	20	X	Good	-	*Picrophloeus*	*belukar*
T2	21	X	Poor	-	*Acacia*	*mangium*
T2	22	X	Poor	-	*Acacia*	*mangium*
T2	23	/	Poor	Medium	*Acacia*	*mangium*
T2	24	/	Poor	Low	*Acacia*	*mangium*
T2	25	/	Fair	Medium	*Acacia*	*mangium*
T2	26	/	Fair	Medium	*Acacia*	*mangium*
T2	27	/	Fair	Medium	*Acacia*	*mangium*
T2	28	/	Fair	Low	*Acacia*	*mangium*
T2	29	X	Fair	-	*Acacia*	*mangium*
T2	30	X	Fair	-	*Acacia*	*mangium*
T2	31	/	Poor	Low	*Acacia*	*mangium*
T2	32	X	Poor	-	*Acacia*	*mangium*
T2	33	X	Poor	-	*Acacia*	*mangium*
T2	34	X	Fair	-	*Acacia*	*mangium*
T2	35	X	Fair	-	*Acacia*	*mangium*
T2	36	X	Fair	-	*Acacia*	*mangium*
T2	37	X	Fair	-	*Acacia*	*mangium*
T2	38	X	Fair	-	*Acacia*	*mangium*
T2	39	X	Fair	-	*Acacia*	*mangium*
T2	40	X	Fair	-	*Acacia*	*mangium*
T2	41	X	Poor	-	*Acacia*	*mangium*
T2	42	X	Poor	-	*Acacia*	*mangium*
T2	43	X	Good	-	*Acacia*	*mangium*
T2	44	X	Good	-	*Acacia*	*mangium*
T2	45	X	Fair	-	*Acacia*	*mangium*
T2	46	X	Good	-	*Acacia*	*auriculiformis*
T2	47	X	Good	-	*Acacia*	*mangium*
T2	48	X	Fair	-	*Acacia*	*mangium*
T2	49	X	Fair	-	*Acacia*	*mangium*
T2	50	X	Poor	-	*Acacia*	*mangium*
T2	51	X	Good	-	*Elaeocarpus*	*mastersii*
T2	52	/	Fair	Medium	*Acacia*	*auriculiformis*
T2	53	/	Good	Low	*Melastoma*	*malabathricum*
T2	54	X	Poor	-	*Acacia*	*mangium*
T2	55	X	Fair	-	*Acacia*	*mangium*
T2	56	X	Fair	-	*Pternandra*	*coerulescens*
T2	57	X	Good	-	*Buchanania*	*arborescens*
T2	58	X	Good	-	*Acacia*	*mangium*
T2	59	/	Fair	Low	*Psychotria*	*sarmentosa*
T2	60	X	Fair	-	*Acacia*	*mangium*
T2	61	X	Fair	-	*Acacia*	*mangium*
T2	62	X	Fair	-	*Acacia*	*mangium*
T2	63	X	Fair	-	*Timonius*	*flavescens*
T2	64	X	Fair	-	*Psychotria*	*sarmentosa*
T2	65	X	Fair	-	*Acacia*	*mangium*
T2	66	/	Fair	Low	*Timonius*	*flavescens*
T2	67	X	Fair	-	*Acacia*	*mangium*
T2	68	X	Fair	-	*Melastoma*	*malabathricum*
T2	69	X	Fair	-	*Melastoma*	*malabathricum*
T2	70	X	Good	-	*Endospermum*	*diadenum*
T2	71	/	Good	Low	*Buchanania*	*arborescens*
T2	72	X	Good	-	*Acacia*	*mangium*
T2	73	X	Good	-	*Timonius*	*flavescens*
T2	74	X	Good	-	*Dillenia*	*suffruticosa*
T2	75	X	Fair	-	*Acacia*	*mangium*
T2	76	X	Good	-	*Timonius*	*flavescens*
T2	77	X	Good	-	*Timonius*	*flavescens*
T2	78	/	Good	Low	*Melastoma*	*malabathricum*
T2	79	X	Fair	-	*Melastoma*	*malabathricum*
T2	80	X	Good	-	*Timonius*	*flavescens*
T2	81	X	Good	-	*Melastoma*	*malabathricum*
T3	1	X	Good	-	*Dillenia*	*suffruticosa*
T3	2	/	Fair	Low	*Vitex*	*pinnata*
T3	3	/	Good	Medium	*Dillenia*	*suffruticosa*
T3	4	/	Good	Very high	*Melastoma*	*malabathricum*
T3	5	/	Good	Medium	*Melastoma*	*malabathricum*
T3	6	/	Fair	Low	*Comersonia*	*batramia*
T3	7	X	Good	-	*Melastoma*	*malabathricum*
T3	8	X	Good	-	*Melastoma*	*malabathricum*
T3	9	X	Good	-	*Melastoma*	*malabathricum*
T3	10	X	Fair	-	*Comersonia*	*batramia*
T3	11	/	Good	Very high	*Comersonia*	*batramia*
T3	12	/	Good	Medium	*Macaranga*	*tanarius*
T3	13	X	Fair	-	*Comersonia*	*batramia*
T3	14	X	Good	-	*Dillenia*	*suffruticosa*
T3	15	/	Good	Low	*Acacia*	*mangium*
T3	16	X	Good	-	*Melastoma*	*malabathricum*
T3	17	X	Good	-	*Dillenia*	*suffruticosa*
T3	18	/	Good	Low	*Melastoma*	*malabathricum*
T3	19	X	Good	-	*Melastoma*	*malabathricum*
T3	20	X	Good	-	*Melastoma*	*malabathricum*
T3	21	X	Good	-	*Melastoma*	*malabathricum*
T3	22	X	Good	-	*Melastoma*	*malabathricum*
T3	23	X	Good	-	*Melastoma*	*malabathricum*
T3	24	X	Good	-	*Melastoma*	*malabathricum*
T3	25	X	Good	-	*Vitex*	*pinnata*
T3	26	X	Good	-	*Acacia*	*auriculiformis*
T3	27	X	Good	-	*Acacia*	*auriculiformis*
T3	28	X	Good	-	*Melastoma*	*malabathricum*
T3	29	X	Good	-	*Melastoma*	*malabathricum*
T3	30	X	Good	-	*Melastoma*	*malabathricum*
T3	31	X	Good	-	*Melastoma*	*malabathricum*
T3	32	X	Good	-	*Melastoma*	*malabathricum*
T3	33	X	Good	-	*Dillenia*	*suffruticosa*
T3	34	/	Good	Low	*Melastoma*	*malabathricum*
T3	35	X	Good	-	*Dillenia*	*suffruticosa*
T3	36	X	Good	-	*Dillenia*	*suffruticosa*
T3	37	X	Good	-	*Dillenia*	*suffruticosa*
T3	38	X	Good	-	*Dillenia*	*suffruticosa*
T3	39	X	Good	-	*Dillenia*	*suffruticosa*
T3	40	X	Good	-	*Dillenia*	*suffruticosa*
T3	41	X	Good	-	*Dillenia*	*suffruticosa*
T3	42	X	Good	-	*Dillenia*	*suffruticosa*
T3	43	X	Good	-	*Dillenia*	*suffruticosa*
T3	44	X	Good	-	*Acacia*	*mangium*
T3	45	X	Good	-	*Vitex*	*pinnata*
T3	46	/	Good	High	*Nepenthes*	*gracilis*
T3	47	/	Good	High	*Nepenthes*	*gracilis*
T3	48	/	Good	High	*Nepenthes*	*gracilis*
T3	49	/	Good	Medium	*Nepenthes*	*gracilis*
T3	50	X	Good	-	*Nepenthes*	*gracilis*
T3	51	/	Good	Low	*Nepenthes*	*gracilis*
T3	52	/		Medium	*Rhodomyrtus*	*tomentosa*
T4	1	/	Good	Low	*Acacia*	*auriculiformis*
T4	2	X	Good	-	*Glochidion*	*littorale*
T4	3	/	Fair	Medium	*Acacia*	*auriculiformis*
T4	4	X	Good	-	*Melastoma*	*malabathricum*
T4	5	X	Good	-	*Vitex*	*pinnata*
T4	6	X	Good	-	*Dillenia*	*suffruticosa*
T4	7	X	Good	-	*Acacia*	*mangium*
T4	8	/	Good	Low	*Acacia*	*mangium*
T4	9	X	Good	-	*Vitex*	*pinnata*
T4	10	X	Good	-	*Vitex*	*pinnata*
T4	11	/	Good	High	*Vitex*	*pinnata*
T4	12	/	Fair	High	*Vitex*	*pinnata*
T4	13	/	Good	Low	*Vitex*	*pinnata*
T4	14	X	Good	-	*Acacia*	*mangium*
T4	15	X	Good	-	*Endospermum*	*diadenum*
T4	16	X	Good	-	*Dillenia*	*suffruticosa*
T4	17	X	Good	-	*Acacia*	*mangium*
T4	18	X	Fair	-	*Vitex*	*pinnata*
T4	19	X	Good	-	*Melastoma*	*malabathricum*
T4	20	/	Good	Low	*Melastoma*	*malabathricum*
T4	21	X	Good	-	*Acacia*	*mangium*
T4	22	X	Good	-	*Melastoma*	*malabathricum*
T4	23	/	Fair	Low	*Endospermum*	*diadenum*
T4	24	X	Good	-	*Acacia*	*mangium*
T4	25	X	Good	-	*Cocos*	*nucifera*
T5	1	/	Good	High	*Melastoma*	*malabathricum*
T5	2	/	Good	Low	*Acacia*	*auriculiformis*
T5	3	X	Good	-	*Acacia*	*auriculiformis*
T5	4	X	Good	-	*Acacia*	*auriculiformis*
T5	5	X	Good	-	*Acacia*	*auriculiformis*
T5	6	X	Good	-	*Alpinia*	*aquatica*
T5	7	X	Good	-	*Melastoma*	*malabathricum*
T5	8	X	Good	-	*Melastoma*	*malabathricum*
T5	9	X	Good	-	*Dilennia*	*suffruticosa*
T5	10	X	Good	-	*Dilennia*	*suffruticosa*
T5	11	X	Good	-	*Dilennia*	*suffruticosa*
T5	12	X	Good	-	*Melastoma*	*malabathricum*
T5	13	X	Good	-	*Dilennia*	*suffruticosa*
T5	14	X	Good	-	*Melastoma*	*malabathricum*
T5	15	X	Good	-	*Melastoma*	*malabathricum*
T5	16	X	Good	-	*Melastoma*	*malabathricum*
T5	17	X	Good	-	*Elaeocarpus*	*aff. mastersii*
T5	18	X	Good	-	*Melastoma*	*malabathricum*
T5	19	X	Good	-	*Melastoma*	*malabathricum*
T5	20	X	Good	-	*Dilennia*	*suffruticosa*
T5	21	X	Good	-	*Melastoma*	*malabathricum*
T5	22	X	Good	-	*Melastoma*	*malabathricum*
T5	23	X	Good	-	*Acacia*	*auriculiformis*
T5	24	X	Good	-	*Melastoma*	*malabathricum*
T5	25	X	Good	-	*Melastoma*	*malabathricum*
T5	26	X	Good	-	*Melastoma*	*malabathricum*
T5	27	/	Good	Low	*Dilennia*	*suffruticosa*
T5	28	/	Good	Medium	*Acacia*	*auriculiformis*
T5	29	X	Good	-	*Elaeocarpus*	*marginatus*
T6	1	X	Fair	-	*Acacia*	*auriculiformis*
T6	2	X	Fair	-	*Acacia*	*auriculiformis*
T6	3	/	Good	Low	*Casuarina*	*equisetifolia*
T6	4	X	Good	-	*Melastoma*	*malabathricum*
T6	5	X	Good	-	*Dilennia*	*suffruticosa*
T6	6	X	Fair	-	*Acacia*	*auriculiformis*
T6	7	X	Poor	-	*Acacia*	*mangium*
T6	8	X	Fair	-	*Acacia*	*mangium*
T6	9	X	Fair	-	*Acacia*	*auriculiformis*
T6	10	X	Good	-	*Dilennia*	*suffruticosa*
T6	11	X	Good	-	*Dilennia*	*suffruticosa*
T6	12	X	Good	-	*Dilennia*	*suffruticosa*
T6	13	X	Good	-	*Dilennia*	*suffruticosa*
T6	14	X	Good	-	*Dilennia*	*suffruticosa*
T6	15	X	Fair	-	*Acacia*	*mangium*
T6	16	X	Good	-	*Acacia*	*mangium*
T6	17	X	Good	-	*Dilennia*	*suffruticosa*
T6	18	/	Fair	Low	*Dilennia*	*suffruticosa*
T6	19	/	Fair	High	*Acacia*	*auriculiformis*
T6	20	/	Fair	Low	*Acacia*	*mangium*
T6	21	/	Fair	Medium	*Dilennia*	*suffruticosa*
T6	22	/	Good	Medium	*Melastoma*	*malabathricum*
T6	23	/	Fair	Low	*Acacia*	*mangium*
T6	24	/	Fair	High	*Acacia*	*auriculiformis*
T6	25	/	Fair	High	*Acacia*	*auriculiformis*
T6	26	/	Good	Medium	*Melastoma*	*malabathricum*
T6	27	/	Good	Low	*Dilennia*	*suffruticosa*
T6	28	/	Fair	High	*Acacia*	*auriculiformis*
T6	29	/	Good	Low	*Dilennia*	*suffruticosa*
T6	30	/	Fair	V.High	*Acacia*	*auriculiformis*
T6	31	/	Good	V.High	*Acacia*	*auriculiformis*
T6	32	/	Fair	Medium	*Acacia*	*auriculiformis*
T6	33	/	Fair	Low	*Acacia*	*auriculiformis*
T6	34	X	Fair	-	*Dilennia*	*suffruticosa*
T6	35	X	Good	-	*Melastoma*	*malabathricum*
T6	36	/	Fair	Medium	*Dilennia*	*suffruticosa*
T6	37	/	Good	Low	*Dilennia*	*suffruticosa*
T6	38	/	Good	h	*Acacia*	*auriculiformis*
T6	39	/	Good	Low	*Dilennia*	*suffruticosa*
T6	40	/	Fair	Low	*Dilennia*	*suffruticosa*
T6	41	X	Fair	-	*Dilennia*	*suffruticosa*
T6	42	X	Fair	-	*Dilennia*	*suffruticosa*
T6	43	/	Good	Low	*Dilennia*	*suffruticosa*
T6	44	/	Good	Low	*Dilennia*	*suffruticosa*
T6	45	/	Good	Low	*Dilennia*	*suffruticosa*
T6	46	/	Good	Medium	*Dilennia*	*suffruticosa*
T6	47	X	Good	-	*Dilennia*	*suffruticosa*
T6	48	/	Good	Low	*Dilennia*	*suffruticosa*
T6	49	X	Fair	-	*Dilennia*	*suffruticosa*
T6	50	/	Good	Low	*Dilennia*	*suffruticosa*
T6	51	X	Good	-	*Dilennia*	*suffruticosa*
T6	52	/	Good	Low	*Dilennia*	*suffruticosa*
T6	53	X	Fair	-	*Dilennia*	*suffruticosa*
T6	54	X	Good	-	*Dilennia*	*suffruticosa*
T6	55	X	Fair	-	*Dilennia*	*suffruticosa*
T6	56	X	Fair	-	*Dilennia*	*suffruticosa*
T6	57	X	Good	-	*Dilennia*	*suffruticosa*
T6	58	X	Good	-	*Dilennia*	*suffruticosa*
T6	59	X	Fair	-	*Dilennia*	*suffruticosa*
T6	60	X	Good	-	*Dilennia*	*suffruticosa*
T6	61	X	Good	-	*Dilennia*	*suffruticosa*
T6	62	X	Good	-	*Dilennia*	*suffruticosa*
T6	63	X	Fair	-	*Dilennia*	*suffruticosa*
T6	64	/	Good	Medium	*Dilennia*	*suffruticosa*
T6	65	/	Good	Low	*Dilennia*	*suffruticosa*
T6	66	X	Good	-	*Dilennia*	*suffruticosa*
T6	67	X	Fair	-	*Dilennia*	*suffruticosa*
T6	68	X	Fair	-	*Dilennia*	*suffruticosa*
T6	69	X	Good	-	*Dilennia*	*suffruticosa*
T6	70	/	Good	Low	*Dilennia*	*suffruticosa*
T6	71	/	Good	High	*Dilennia*	*suffruticosa*
T6	72	X	Good	-	*Dilennia*	*suffruticosa*
T6	73	/	Good	Low	*Dilennia*	*suffruticosa*
T6	74	/	Fair	Medium	*Acacia*	*mangium*
T6	75	X	Good	-	*Acacia*	*mangium*
T6	76	X	Good	-	*Acacia*	*mangium*
T6	77	X	Good	-	*Dilennia*	*suffruticosa*
T6	78	X	Fair	-	*Acacia*	*mangium*
T6	79	/	Good	Medium	*Dilennia*	*suffruticosa*
T6	80	X	Good	-	*Acacia*	*mangium*
T6	81	X	Good	-	*Casuarina*	*equisetifolia*
T6	82	X	Good	-	*Casuarina*	*equisetifolia*
T6	83	/	Good	Medium	*Casuarina*	*equisetifolia*
T6	84	X	Good	-	*Casuarina*	*equisetifolia*
T6	85	X	Good	-	*Casuarina*	*equisetifolia*
